# Agri-Food Wastes as Natural Source of Bioactive Antioxidants

**DOI:** 10.3390/antiox12020351

**Published:** 2023-02-02

**Authors:** Silvana Hrelia, Cristina Angeloni, Maria Cristina Barbalace

**Affiliations:** Department for Life Quality Studies, Alma Mater Studiorum, University of Bologna, Corso d’Augusto 237, 47921 Rimini, RN, Italy

Nowadays, the health of the ecosystem and quality of life are jeopardized by the growing quantities of waste that are released into the environment. At a global level, the agri-food industries are among the major producers of waste [1]. It should be understood that, every year, Europe alone is responsible for wasting around 90 million tons of food and 700 million tons of crops [2]. Agri-food by-products are derived from skins, peels, seeds, leaves, and other inedible elements that are usually underutilized and cause serious problems for the environment. These wastes are rich in organic matter and other elements that modify the composition of the soils and the streams where they are disposed, causing, for example, the death of aquatic organisms [3]. Another problem is related to the habit of burning agri-food waste that may generate several toxic compounds [4]. According to the United Nations, the market value of food lost or wasted worldwide is approximately USD 936 billion. Another very important aspect is that these wastes are responsible for 8% of all annual global greenhouse gas emissions [5]. In recent years, many studies have shown that agri-food by-products are rich in precious compounds that possess potential bioactivity [6,7,8,9]. In fact, agri-food by-products are characterized by the presence of polysaccharides, proteins, carbohydrates, polyphenolic constituents, etc. [10]. For these reasons, they are valuable renewable natural resources with the added advantage of being inexpensive, easy to access, eco-friendly, and sustainable. At present, the circular economy offers various tools to recover waste, and one of the best alternatives includes transforming them into high-commercial-value products, such as drugs, nutraceuticals, and cosmeceuticals [11,12]. To effectively recover agri-food waste, it is necessary to develop green and sustainable alternatives to conventional extraction methods to increase the extraction yield and decrease the extraction time and solvent consumption.

This Special Issue on the use of agri-food wastes as a natural source of bioactive antioxidants contains 18 contributions: 15 research articles and 3 reviews, addressing the most recent advances on this topic.

Different research papers of this Special Issue focused on agri-food by-products derived from fruit and vegetables typical of the Mediterranean diet: grape, apple, orange, tomato, allium, and pea.

Grapes (*Vitis vinifera)* can be considered one of the major fruit crops around the world and, as a consequence, huge quantities of wastes are produced during its processing, especially for the production of wine and juices. In this Special Issue, five papers focused their attention on the analysis of the phytochemical composition and biological activities of wastes derived from different varieties of grapes. Among grape varieties, muscadine grapes (*Vitis rotundofilia*) are known to possess a high amount of antioxidant compounds. For these reasons, several muscadine grapes supplements, mainly composed of ground dried skin and grape seeds, are already commercially available. Unfortunately, information regarding the phenolic composition of muscadine grapes supplements is really limited. The study from Chappell et al. [13] investigated the phenolic profiles, antioxidant activity, and protective effects against angiotensin II in H9c2 cardiomyocytes of four commercially processed muscadine grapes supplements. The results demonstrated that the dried water-extract muscadine grape supplement was the richest in epicatechin, ellagic acid, gallic acid, procyanidin B2, catechin, and catechin gallate, and in total phenolic content compared to the other three supplements. Accordingly, only this supplement was able to counteract the increase in malondialdehyde and 4-hydroxynonanol levels and to boost the activity of superoxide dismutase 1 and catalase enzymes in H9c2 cells. This study also demonstrated that muscadine grapes supplements can be formulated commercially thanks to the stability and consistent results among different lots analyzed.

Dabetic et al. [14] focused on the optimization of an extraction method with the aim to obtain the highest phenolic content from the seeds of eight red grape varieties. In recent years, growing attention has been paid to the development of extraction methods with ‘green’ potential, so the authors compared the efficiency of a conventional solvent, such as ethanol, to a green solvent, such as ChCit—choline chloride:citric acid. We found that the best extraction conditions were: a sample:solvent ratio of 1:10 *w*/*v*, extraction time of 30 min, and extraction temperature of 50 °C, as measured by HPLC–MS/MS analyses. Moreover, the use of ChCit solvent had fascinating results, including a better correlation between phenolic content and antioxidant activity evaluated by DPPH, FRAP, ABTS, and CUPRAC assays.

Herrera-Bravo et al. [15] evaluated the activity of anthocyanins found in Pinot noir pomace extract on Nrf2 signaling in endothelial cells. Malvidin-3-glucoside was the main anthocyanin found in Pinot noir pomace, and with docking studies, the authors demonstrated the ability of this compound to interact with the activation of Nrf2. Indeed, the treatment of endothelial cells with Pinot noir pomace extract led to the up-regulation of HO-1 and NQO1 antioxidant genes, known to be downstream targets of Nrf2 signaling. These results evidenced Pinot noir pomace as a source of bioactive molecules with potential cardioprotective activity.

Della Vedova et al. [16] studied the phenolic composition and the biological activity of four commercial enocianinas, grape pomace extracts particularly rich in anthocyanins. By applying LC-HRMS analysis, the authors found that enocianina extracts were significantly rich in polyphenols, but the anthocyanins represented only a minor fraction. Furthermore, the antioxidant capacity assessed by the DPPH assay evidenced a correlation with the total phenolic content and not with anthocyanins. Regarding biological activities, all extracts demonstrated to dose-dependently activate the Nrf2 signaling pathway. At the same time, all extracts exerted anti-inflammatory effects and such activity was strictly related to the phenolic composition but also to the presence of non-catechol polyphenols.

Al-Warhi et al. [17] investigated the potential of a *Vitis vinifera* seed extract on wound repair in rats. The wound healing analyses revealed a marked repairing activity through accelerating the wound closure, increasing the expression of genes and proteins related to the healing process, including transforming growth factor β1 (TGF-β1), Type I collagen, and vascular endothelial growth factor (VEGF), and, at the same time, reducing the expression of inflammatory cytokines, such as tumor necrosis factor-α (TNF-α) and interleukin-1β (IL-1β). To unravel the possible mechanisms behind the wound-healing activity, the authors applied molecular docking analysis on three molecular targets (TNF-α, TGFBR1, and IL-1β). Additionally, the radicals’ scavenging activity of seed extract and two isolated compounds (ursolic acid and β-sitosterol-3-O-glucopyranoside) was evaluated, indicating the strong antioxidant capacity of the whole extract with respect to the single compounds. From this study, we can conclude that the application of *Vitis vinifera* seed extract has great potential in wound care therapy, though further analyses are needed.

The Annurca apple is native to Southern Italy, listed as a Protected Geographical Indication (PGI) product by the European Council (Commission Regulation (EC) No.417/2006). The Annurca apple has the ability to control cholesterol plasma levels, which has already been demonstrated [18], but no studies have investigated the composition and bioactivity of Annurca apple leaves. It is noteworthy that, apple and apple by-products are a rich source of phlorizin, a member of dihydrochalcones, whose anti-inflammatory, antioxidant, anticancer, antibacterial, and anti-diabetic activities have been demonstrated [19]. On these bases, Maisto et al. [20] optimized a method to obtain an extract rich in phlorizin from Annurca apple tree leaves using response surface methodology (RSM) and characterized this extract identifying 23 phenolic molecules. The extract was also investigated for its nutraceutical potential using some in vitro assays: DPPH, ABTS, and FRAP assays to study its antioxidant activity, and the inhibition of the production of advanced glycation end products (AGEs) to investigate its potential anti-diabetic activity. The results showed high antioxidant activity and a good inhibition of AGEs formation suggesting this extract as a powerful functional ingredient, useful for the formulation of nutraceutical products for the management of diabetes disease.

Comacho et al. [21] studied the phenol composition and antioxidant activity of orange juice by-product extracts obtained with subsequent extraction to extract both free phenols and phenols bound to plant tissues. In particular, they used four successive extractions: the first two extractions (MeOH 30 °C and MeOH 60 °C) were able to extract free phenols; the last two subsequent basic and acidic extractions yielded the phenols bound to plant tissues. The results demonstrated the importance of using more specific extraction methods than those conventionally used to obtain both the free phenolic compounds and those bound to other cellular structures, representing nearly 20% of the total phenols and having a major role in the antioxidant capacity of the product.

Tomato by-products are mainly composed of skin and seeds and represent between 1.5% and 5% of the initial weight. Tomatoes are a good source of the hydrophobic carotenoid lycopene which possesses several beneficial properties acting as a potent antioxidant [22]. As an important characteristic of the solvents used in the extraction of phytocompounds is their low toxicity, Campos-Lozada et al. [23] used three types of vegetable oils (grape, extra virgin olive, and peanut) by means of two methods (agitation and high-intensity ultrasound) to extract lycopene by tomato by-product. The results demonstrated that the high-intensity ultrasound technique is not suitable for the extraction of lycopene due to the formation of free radicals that oxidize lycopene. On the other hand, the magnetic stirring technique is a good technique due to lower aggressiveness and the absence of free radicals. Peanut oil is not recommended as a solvent in the extraction of lycopene because of its high content of unsaturated fatty acids susceptible to oxidation. Olive oil gave the best results as it extracted the highest quantity of lycopene and represents an optimal solvent thanks to its safety.

Onion is the second most cultivated crop worldwide after tomatoes. Onions are rich in antioxidant compounds and their consumption has been associated with a reduction in the incidence of various degenerative diseases. Every year, huge amounts of bio-waste are generated during onion processing, mainly composed of onion skin. Due to the onion’s unpleasant taste and smell its by-products are not suitable for animal feeding or as organic fertilizer. Chernukha et al. [24] assessed the antioxidant potential of husk waste obtained from red, yellow, and white varieties of onions. Only in red and yellow onion husks flavonols, flavanonols, flavonoid-O-glycosides, and isoflavones were detected. Quercetin and its glucosides were the most representative flavonoids in the onion husks. The highest content of flavonoids was determined in red onion husks, and this was associated with the higher antioxidant activity of the red onion husk ethanol extract.

Pea seeds are characterized by a high nutritional value with a high content of proteins, starch, fibers, minerals, and vitamins. Every year, the pea industry produces a big quantity of by-products, and their recycling is of great importance. Pods are the main pea-waste, which make up about 30–67% of the total weight of the whole pod. In light of these considerations, Castaldo et al. [25] investigated the polyphenolic fraction of a water-based extract of pea pods using an UHPLC-Q-Orbitrap HRMS methodology. The most commonly detected polyphenolic compounds were mainly represented by 5-caffeoylquinic acid, epicatechin, hesperidin, and catechin. Moreover, two different nutraceutical preparations (acid-resistant capsules (ARC) and non-acid-resistant capsules (NARC)) were produced and subjected to simulated GI digestion. The ARC formulations were able to preserve the active compounds along the simulated GI process, highlighting a higher total phenolic compounds value and antioxidant capacity than the NARC formulations.

One paper, marginally related to the topic of the Special Issue, investigated the antioxidant activities and the presence of bioactive constituents in several plant aqueous extracts (*Curcuma longa*; *Myristica fragrans*; *Zingiber officinale*; *Cymbopogon citratus* and *Thymus vulgaris*, as well as their mixture) and also evaluated the effect of these extracts on quality aspects of rabbit meat during 16-day cold storage [26]. The aqueous extract of *Myristica fragrans* had the highest ABTS scavenging activity, while *Zingiber officinale* showed the highest DPPH scavenging activity. Eugenol was the most abundant compound in *Curcuma longa*. *Myristica fragrans* was rich in cinnamaldehyde, γ-terpinene, and 4-Allyl-2-methoxy phenol. *Zingiber officinale* extract was characterized by the presence of thymol, methylheptenone, and tricycle. Eicosane aldehyde, caryophyllene, octadecanoic acid, and hexadecenoic acid were the main compounds in *Cymbopogon citratus* extract. Finally, the major components of *Thymus vulgaris* were thymol, p-cymene, and γ-terpineol. The treatment of cold-stored rabbit meat with 0.2% of the extract’s mixture doubled the storage time with acceptable odor and taste suggesting that plant extracts may be effective against rancidity and may be used as a natural antioxidant to increase the shelf life of cold-stored rabbit meat.

In this Special Issue, four papers have turned attention to the by-products of some exotic fruits and vegetables which see great popularity on the market.

*Psidium guajava* is a tropical edible fruiting plant and Shady et al. [27] studied the supplementation of *Psidium guajava* seed extracts to rats before a single acute oral dose of indomethacin to induce gastric ulcers. The chemical composition of the crude extract was evaluated by metabolic profiling using HR-LCMS to investigate the chemical constituents that might be responsible for the protective role against ulcer formation, evaluated by histopathological analyses on a rat stomach.

A molecular docking technique was applied to identify potential mechanisms of action of the identified compounds acting as anti-ulcer suggesting a protective role for phytosterols. In vitro analysis demonstrated the antioxidant effect of the extract. The study highlights the possible use of *Psidium guajava* seed extract against ulceration as an alternative to anti-ulcer drugs.

Aloe vera skin is a major by-product of Aloe processing plants and accounts for over 30% of the total leaf weight, generating large amounts of waste. Using microwave-assisted extraction Solaberrieta et al. [28] obtained an interesting extract from Aloe vera skin, with a high phenolic content, mainly represented by aloin A, aloin B, aloesin, aloe-emodin, aloeresin D, orientin, cinnamic acid and chlorogenic acid, characterized and quantified by HPLC-MS and HPLC-DAD, respectively. The reported extraction technique could represent a promising procedure for obtaining antioxidant extracts rich in polyphenols with potential industrial applications.

Avocado seed and peel are the main by-products from avocado, accounting for nearly 30% of fruit weight, and they are usually discarded. A tentative analytical characterization/quantification by HPLC-ESI-qTOF-MS of the phenolic composition of avocado seed and peel was performed by Rojas-Garcia et al. [29], together with an in-depth in vitro study, to evaluate antioxidant potential and free-radical scavenging activity, as well as the inhibitory effect of its phenolics on Acetylcholinesterase, Elastase, Hyaluronidase, and Collagenase. Additionally, an evaluation of human platelet anti-aggregatory potential was performed, evidencing the anti-aggregatory role of avocado peel. This study provided new information about avocado waste treatment from a preindustrial scale-up point of view for those industries that generate many tons of waste per year and want to invest in revalorization of by-products with potential health effects.

Mango (*Mangifera indica* L.) is widely cultivated in tropical and subtropical areas of the world but in recent years, this cultivation has also spread in different regions of the Mediterranean area, such as Sicily (Italy). The edible part of the mango is only the pulp and peel, and seeds are the main bio-wastes representing a consistent part of the fruit. Pratelli et al. [30], starting from lyophilized mango peel and seed, obtained ethanolic extracts that were chemically characterized by HPLC-ESI-MS. The authors evaluated anti-obesity effects of extracts using 3T3-L1 cells. Both peel and seed extracts reduced lipid accumulation and triacylglycerol content by down-regulating the key adipogenic transcription factor PPARγ and its downstream targets and inhibiting the activation of AMPK with the consequent inhibition of Acetyl-CoA-carboxylase. Data obtained suggest that the bio-waste products of mango are promising anti-obesity natural compounds.

The reviews published in this Special Issue are focused on different aspects related to agri-food by-products recovery, to the valorization of their main antioxidants as potential nutraceuticals to counteract chronic/degenerative diseases also paying attention to the genotoxicity risk assessment of bioactive compounds extracted from agricultural waste.

In recent years, significant attention has been paid to sustainable approaches for the extraction of bioactive compounds from agri-food wastes and to the application of green extraction techniques. Lianza et al. [31] provided a comprehensive overview of the most recent green extraction procedures applied for the recovery of anthocyanins from plant-derived wastes and by-products. Water, ethanol, glycerol, and natural deep eutectic solvents were taken into consideration as solvents and ultrasound-assisted extraction, pressurized liquid extraction, microwave-assisted extraction, supercritical fluid extraction, and electric treatments were critically discussed as green techniques. The authors concluded that the effectiveness of anthocyanin extraction is the result of a combination between the solvent used and the applied technique also in light of energy cost, safety, and environmental impact.

Caliceti et al. [32] summarized the current knowledge on the possible exploitation of waste or by-products derived by the processing of three traditional Italian crops, apple, pear, and sugar beet, as a source of bioactive molecules to protect endothelial function. They focused on the chemical profile of these pomaces and on their efficacy in various pathological conditions related to endothelial dysfunction linked to cardiovascular diseases. The review underlines that, when properly processed, these by-products represent a real reservoir of bioactive molecules with a health-promoting action toward endothelial dysfunction and its major complications.

Musto et al. [33] put attention to the safety of biowaste-derived products. They performed a deep analysis of genotoxic risk assessment of oil, tea, rice, fruits, spices, wine, and some others biowastes, demonstrating that at least one of the first steps in the confirmation of their safety was generally accomplished. The authors concluded that the risk assessment analysis should be completed and include all the toxicological studies requested by the authorities. Waste recycling without any risk to humans, animals, or the environment is of great importance and must be guaranteed.

In conclusion, this Special Issue highlights the advantages of recycling agri-food waste by producing nutraceuticals. This virtuous cycle has the advantage of reducing the negative impact of waste products on the environment and allowing the production of nutraceuticals at low cost since they are obtained from low-economic-value waste. Additionally of great importance is the identification of extraction techniques that use solvents with a low environmental impact and that are safe for human beings.
